# Estimating the Least Principal Stress in a Granitic Rock Mass: Systematic Mini-Frac Tests and Elaborated Pressure Transient Analysis

**DOI:** 10.1007/s00603-021-02743-1

**Published:** 2022-01-18

**Authors:** Kai Bröker, Xiaodong Ma

**Affiliations:** Department of Earth Sciences, ETH Zurich, Sonneggstrasse 5, 8092 Zurich, Switzerland

**Keywords:** Bedretto Underground Laboratory, In situ stress measurement, Hydraulic fracturing, Mini-frac test, Pressure transient analysis, Crystalline rock

## Abstract

The hydraulic fracturing technique (also termed mini-frac test) is commonly used to estimate the in situ stress field. We recently conducted a mini-frac stress measurement campaign in the newly-established Bedretto Underground Laboratory (BedrettoLab) in the Swiss Alps. Four vertical boreholes, dedicated for stress characterization of the granitic rock mass, hosted a total of 19 mini-frac test intervals. Systematic pressure transient analysis was performed to carefully estimate the magnitude of the least principal stress ($S_\mathrm {3}$). We compared five different methods (inflection point, bilinear pressure decay rate, tangent, fracture compliance, and jacking pressure) to identify an adequate approach best suited for our test scale and the host rock mass. We found that the methods used to determine the fracture closure pressure underestimate the magnitude of $S_\mathrm {3}$, presumably due to the rapid closure of the hydraulic fracture after shut-in. The most consistent results were found using the inflection point and bilinear pressure decay rate method, which both determine the (instantaneous) shut-in pressure as the proxy for the $S_\mathrm {3}$ magnitude. The determined shut-in pressure, or $S_\mathrm {3}$ magnitude, is $14.6\pm 1.4$ MPa from the inflection point method. This allowed us to further estimate the stress environment around the BedrettoLab, which is transitional between normal and strike-slip faulting. The measured local pore pressures from extended shut-in periods are between 2.0 and 5.6 MPa, significantly below hydrostatic. A combination of drainage, cooling, and the excavation damage zone of the tunnel may have significantly perturbed the in situ stress field in the vicinity of the BedrettoLab.

## Introduction

Many Earth science and subsurface engineering applications require information about the in situ stress state. Among other techniques, hydraulic fracturing (HF) is widely applied as a direct stress measurement technique in boreholes (Haimson and Cornet 2003). It can be applied without prior knowledge of the elastic rock properties (Haimson and Fairhurst 1967, 1969), and samples a more representative rock volume than other methods (e.g. Amadei and Stephansson 1997; Evans et al. 1999; Zoback 2007; Zang and Stephansson 2010; Schmitt et al. 2012). It is also robust and relatively easy to apply. A hydraulic fracturing test with small injected volume is commonly referred to as mini-frac test, to distinguish it from hydraulic fracturing as a reservoir stimulation technique. Since hydraulic fractures propagate perpendicular to the least principal stress ($S_\mathrm {3}$) direction in a homogenous, i.e. non fractured, rock volume (Hubbert and Willis 1957), mini-frac tests give a good estimate of the $S_\mathrm {3}$ magnitude and orientation.

Stress measurements are particularly important for Underground Research Laboratories (URL), which require the rock volume to be well characterized, usually on a scale from 10 to 100 m, to provide a high level of experimental control for in situ experiments. In many URL projects, mini-frac tests have frequently been utilized to determine the stress orientations and magnitudes. Some examples are: an URL for nuclear waste storage in Canada (Martin 1990; Martin et al. 1990), the Jurong caverns in Singapore (Synn et al. 2015), the Korean Underground Research Tunnel for nuclear waste storage in South Korea (Jo et al. 2019), the Grimsel Test Site in Switzerland (Amann et al. 2018; Krietsch et al. 2019), the Äspö Hard Rock Laboratory in Sweden (Ask 2006; Zang et al. 2017), the Sanford Underground Research Facility in South Dakota (US) (Wang et al. 2017; Ingraham et al. 2020; Schoenball et al. 2020), and the Reiche Zeche Underground Laboratory in Germany (Dresen et al. 2019). Mini-frac test results indicate that the in situ stress field can vary significantly at the typical URL scale (e.g. Martin 1990). The stress variations can be driven by local lithological and structural variations, underground opening perturbations, or topographic effects. To fully incorporate the influence of stress variations on in situ experiments, a systematic analysis and evaluation of the mini-frac data are a prerequisite.

Despite the widespread use of mini-frac tests, it is challenging to estimate the magnitude of the least principal stress ($S_\mathrm {3}$) from pressure transient analysis, since there lacks consensus on a standardized data analysis. Various methods have been proposed for different scales, different site conditions, and different applications (e.g., petroleum, civil, and mining engineering). These methods determine the instantaneous shut-in pressure, fracture closure pressure, or jacking pressure as an estimate of the least principal stress magnitude [see Schmitt and Haimson (2017) for a review]. The instantaneous shut-in pressure is a simple and straightforward method to determine $S_\mathrm {3}$ (Haimson and Fairhurst 1969), but there are debates, particularly in the petroleum industry, that it overestimates the $S_\mathrm {3}$ magnitude and the pressure where the hydraulic fracture starts to close (fracture closure pressure) should be used instead (e.g. Economides and Nolte 2000). This issue is exacerbated as the instantaneous shut-in pressure and fracture closure pressure are sometimes used interchangeably (Schmitt and Haimson 2017). Several methods have been compared in previous studies (e.g. Doe and Korbin 1987; Baumgärtner and Zoback 1989; Lee and Haimson 1989; Guo et al. 1993b; Wang and Sharma 2019); however, the choice of the method and the scale of the tests (in terms of injected fluid volume) vary case by case. The applicability of the available methods certainly warrants a systematic evaluation for comparison.

In this paper, we strive to provide such a systematic study in the URL context. We report the results of a series of mini-frac tests recently conducted in the Bedretto Underground Laboratory for Geoenergies and Geosciences (BedrettoLab). We compare five more advanced and frequently used methods to estimate $S_\mathrm {3}$ from these mini-frac tests: inflection point method, bilinear pressure decay rate method, tangent method, fracture compliance method, and the jacking pressure from step-rate injection tests (see Table 1 for references and nomenclature). We focus on assessing which of the methods is most suitable for stress estimation in a granitic rock mass at the URL scale. The results of this study will benefit the planning of future meso-scale in situ experiments at the BedrettoLab.

## Site Description

**Fig. 1:**
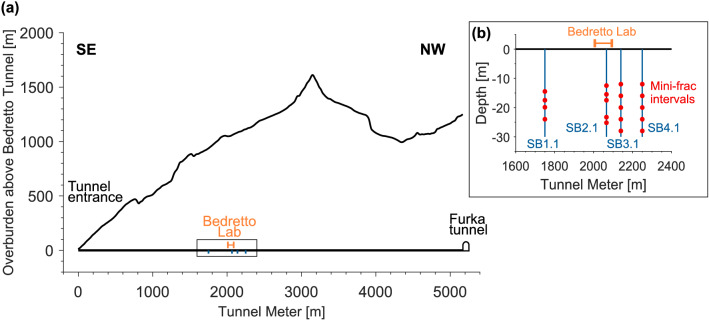
**a** Schematic side view of the Bedretto Tunnel and the topography above. The location of the BedrettoLab is denoted by an orange bar. **b** Close-up of the SB borehole locations and mini-frac intervals therein

The Bedretto Underground Laboratory for Geoenergies and Geosciences (BedrettoLab) is located about 2 km from the entrance of the Bedretto Tunnel, which is an 5.2 km auxiliary access drift of the Furka Base Tunnel. The Bedretto Tunnel is completely unsealed, has a width and height of about 3 m, and its width is enlarged to 6 m at the 100 m-long niche of the BedrettoLab. The topography above the Bedretto Tunnel varies significantly from the southern portal along the Bedretto Tunnel and is about 1030 m at the location of the BedrettoLab (Fig. 1). Future experiments at the BedrettoLab will focus on hydraulic stimulation techniques for enhanced geothermal systems (EGS) and on fault reactivation associated with induced seismicity [see Ma et al. (2021) for more details]. The host rock of the BedrettoLab is the Rotondo Granite intrusion in the Gotthard Massif, which took place during the late stages of the Variscan orogeny (Sergeev et al. 1995). The Rotondo Granite is relatively homogeneous, massive light gray and shows only slight influence of metamorphism (Labhart 2005; Lützenkirchen and Löw 2011). Subvertically dipping fractures and fault zones are developed and intersect with the Bedretto Tunnel, predominantly striking NE–SW to ENE–WSW (Lützenkirchen 2002; Lützenkirchen and Löw 2011). Additionally, steeply dipping fracture zones, striking N–S and E–W, occur frequently along the tunnel (Jordan 2019).

Between December 2018 and July 2019, we conducted mini-frac tests in a total of 19 intervals spanning four vertical boreholes (SB) dedicated for stress measurements (Fig. 1). These boreholes have a diameter of 101 mm and a length of 30 m. At this depth, tests away from the stress concentration around the tunnel are possible to measure the undisturbed in situ stress state. All four boreholes are located along the longitudinal axis of the Bedretto Tunnel and their location is expressed in the unit ‘tunnel meter’ (TM) measured from the southern tunnel entrance. These boreholes are: SB1.1-TM1750, SB2.1-TM2066, SB3.1-TM2250, and SB4.1-TM2140, encompassing the current BedrettoLab between TM2000 and 2100 (Fig. 1). The stress measurements conducted therein allow for the estimation of the stress heterogeneity in the vicinity of the BedrettoLab.

The regional stress field in the Swiss Alps exhibits a transition from a slight predominant strike-slip mechanism in the North Alpine Foreland towards a strong predominance of normal faulting in the high parts of the Alps (Kastrup et al. 2004). The maximum horizontal stress ($S_\mathrm {Hmax}$) direction in the Swiss Alps lies generally within the NW quadrant (Heidbach et al. 2018). Thrust faulting is unlikely in the study area. This is supported by stress-induced failures (e.g., spalling and kinking) observed along sections of the Bedretto Tunnel, indicating that the horizontal stress component perpendicular to the tunnel is smaller than the vertical stress (Gischig et al. 2020; Ma et al. 2020).

It is likely that the tectonic stress field competes with topographic effects from the varying overburden above the Bedretto Tunnel, which can cause significant local stress variations along the tunnel. The overburden of the tunnel gradually rises to its maximum at approximately TM3100 (Fig. 1). For the range of the SB boreholes, the overburden increases from about 940 m at TM1750 to about 1110 m at TM2250. We estimate the average magnitude of the vertical stress ($S_\mathrm {v}$) exerted by the overburden to be about 26.5 MPa by assuming a thickness of the overlying granite of 1000–1050 m with a bulk density of ${2.62}\,\hbox {g cm}^{-3}$ from laboratory measurements (David et al. 2020). A previous study by Meier (2017) showed that the topographic effect diminishes with increasing overburden, and one principal stress direction can be assumed vertical behind approximately TM1500.

## Mini-Frac Test Procedure

**Fig. 2:**
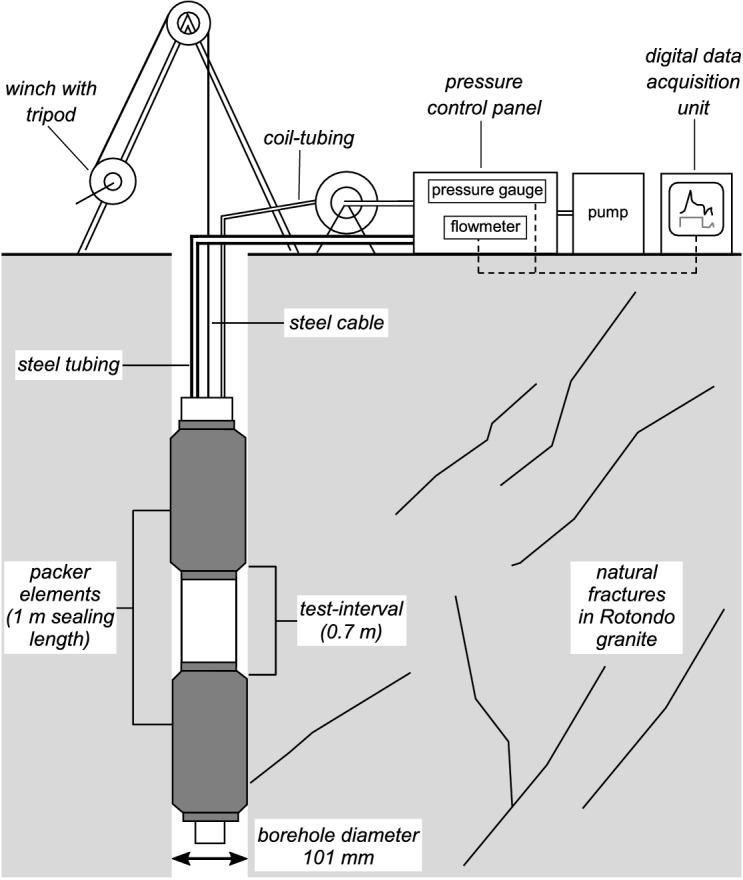
Schematic drawing of the mini-frac equipment and set up (adapted after Klee et al. 2011). Not to scale

The mini-frac tests were carried out using a wireline system (e.g. Klee et al. 2011) with two Kevlar-reinforced straddle packer elements (outer diameter: 91 mm) of 40 MPa pressure capacity (Fig. 2). Each packer has a sealing length of 1 m and the isolated test interval length between them was set to 0.7 m. The packer and interval pressures were measured with electric pressure transducers (KELLER, type PAA-33X; pressure range 0–40 MPa), and the injection rate was measured with a flowmeter (RCI turbine type flowmeter, type QPT01; flow rate range 0–10 $\text { L min}^{-1}$). All pressure and flow rate measurements were made at the surface in the open tunnel. The pressure values reported throughout the paper, however, refer to the ‘downhole’ values, which include the calculated hydrostatic water column pressure at the packer or interval depth. The data were recorded using a digital data acquisition system with a sampling rate of 5–10 Hz, and an electric driven pump (SPECK, type HP 400/1–10; maximum injection rate: ${10}\text { L min}^{-1}$, maximum pressure: 40 MPa) was used for interval and packer pressurization. The surface pump was connected to the downhole packer tool by two separate pressure lines: an 4.5 mm inner diameter stainless steel coil-tubing for packer pressurization and an 11 mm inner diameter steel tubing for injection interval pressurization.

**Fig. 3:**
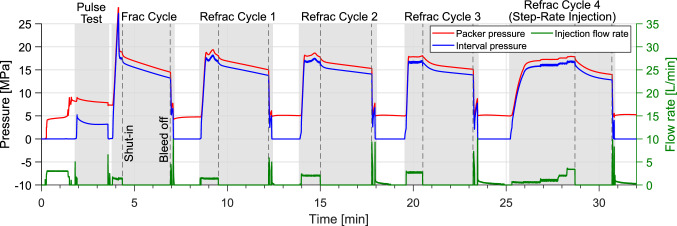
Injection protocol of the mini-frac test in borehole SB3.1 at 12 m depth. The gray shaded sections correspond to different injection cycles, and the dashed lines to pump shut-in and bleed off. Figure 4 shows the frac and part of the first refrac cycle in more detail

Before carrying out the mini-frac tests, we used optical and acoustic televiewer logs to identify sealed and non-sealed pre-existing natural fractures that intersect the boreholes. The orientation (strike and dip) of the natural fractures was analyzed using the software WellCAD. Besides the natural fractures, no stress induced borehole damage such as borehole breakouts or drilling induced tensile fractures were observed. We determined suitable mini-frac intervals free of visible natural fractures based on the pre-frac logging data. In total, 19 tests were conducted in the four vertical boreholes. A standard injection protocol in close agreement with the ISRM suggested method (Haimson and Cornet 2003) was followed (Fig. 3). The protocol started with a pulse test to evaluate packer integrity and whether the interval was sealed off properly. Afterwards, the hydraulic fracture was generated and fully developed during the initial breakdown or ‘frac’ cycle, which was followed by subsequent reopening and propagation in several ‘refrac’ cycles. The injection flow rate was kept constant during each cycle and slightly increased with advancing cycles, varying from 1 to 3 $\text { L min}^{-1}$. We increased the flow rate to compensate for pressure losses and leak-off during fracture propagation. The total injected volume was in the order of 10–25 L per interval. Each injection cycle terminated with a shut-in phase, during which the interval pressure decays due to fluid leak-off into the formation. After the shut-in phase, the interval was vented and the whole hydraulic pressure released, which is referred to as bleed-off.

In each interval, one of the last cycles was conducted as flow rate controlled step-rate injection. These step-rate injections consisted of three to five progressively increasing flow rates to measure the fracture normal stress, which is commonly referred to as jacking pressure ($P_\mathrm {jacking}$). Another modification of the injection protocol was that we extended some shut-in phases, which usually lasted about 3 min, up to 15 min to facilitate the pressure transient analysis. In at least one interval per borehole, we further extended the shut-in to 1 h and even overnight (13–15 h).

In addition to the pre-frac logging, we conducted post-frac logging. We used the logs to determine the hydraulic fracture traces at the borehole wall (location and orientation) and, if any, reactivated natural fractures. This is to ensure that the hydraulic fractures were generated and to evaluate the quality of the test (e.g., fracture growth out of the interval). All but one of the mini-frac tests performed were confirmed by successful fracture generation, providing the basis of the pressure analysis detailed below.

## Determination of Relevant Pressure Parameters from Pressure-Time Records

**Fig. 4:**
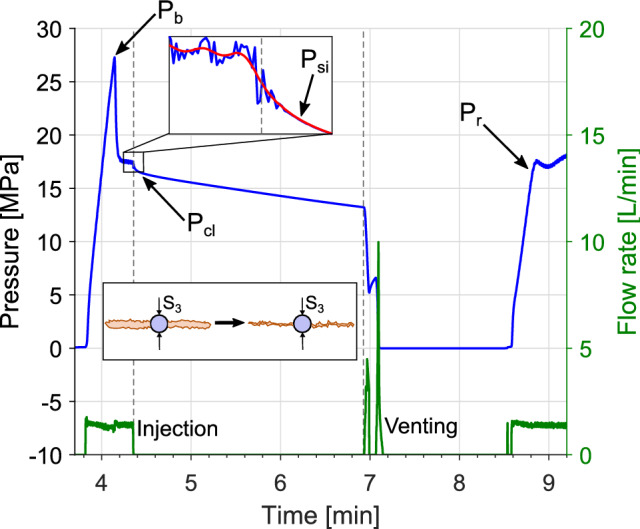
Section of the initial breakdown and first refrac cycle of the mini-frac test at 12 m in SB3.1 (complete injection protocol in Fig. 3). The pressure parameters are: $P_\mathrm {b}$ = formation breakdown pressure, $P_\mathrm {si}$ = (instantaneous) shut-in pressure, $P_\mathrm {cl}$ = fracture closure pressure, $P_\mathrm {r}$ = fracture reopening pressure. The upper close-up shows the unfiltered data with cyclic oscillations upon abrupt shut-in and the filtered data. The lower sketch shows how the hydraulic fracture propagates away from the borehole perpendicular to the least principal stress and how it closes on rough walls once the pressure falls below the closure pressure

**Fig. 5:**
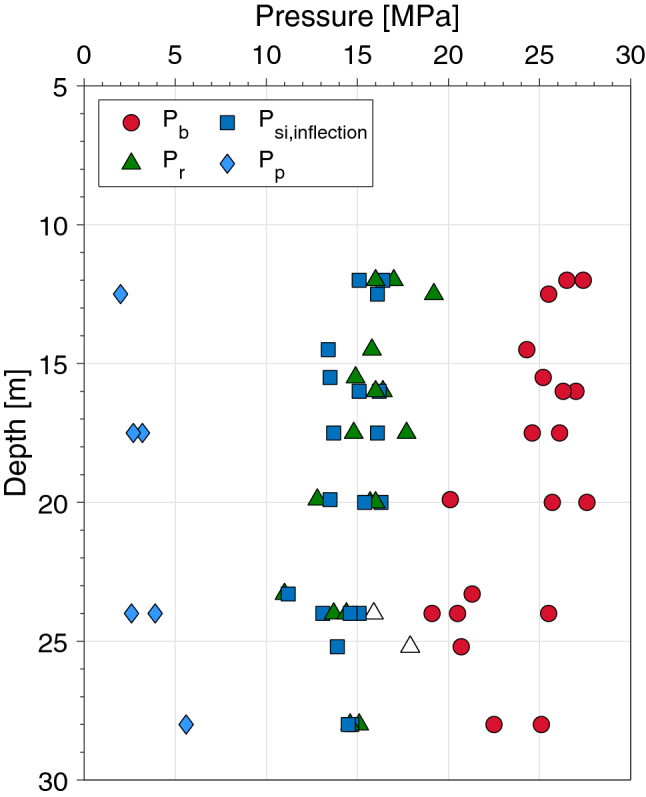
Overview of the mini-frac results of all four vertical boreholes. $P_\mathrm {b}$ denotes breakdown pressure and $P_\mathrm {r}$ denotes reopening pressure. Open symbols indicate unreliable estimates. The shut-in pressure ($P_\mathrm {si,inflection}$) was determined by the inflection point method; the mean values of the first five cycles of each interval are shown. The pore pressure ($P_\mathrm {p}$) was measured by extended shut-in periods (see Fig. 8)

In the following paragraphs, we describe how we estimated the relevant pressure values for in situ stress analysis, give the range of their variations, and provide a limited discussion. A typical interval pressure trend during a mini-frac test is shown in Fig. 4 using a real data example. We picked the following pressures: formation breakdown pressure ($P_\mathrm {b}$), instantaneous shut-in pressure ($P_\mathrm {si}$, or commonly abbreviated as ISIP), fracture closure pressure ($P_\mathrm {cl}$), and hydraulic reopening pressure ($P_\mathrm {r}$). Table 2 summarizes the estimated pressure values of all four SB boreholes, and Fig. 5 summarizes the results described in this section.

### Breakdown Pressure

The formation breakdown pressure ($P_\mathrm {b}$) corresponds to the maximum pressure during the initial cycle of each interval (Fig. 4). This pressure peak corresponds to the unstable propagation of the tensile fracture, which means that the fracture volume increases more rapidly than can be sustained by flow into it from the pump, resulting in a pressure drop (Rummel 1987; Guo et al. 1993a; Bunger et al. 2010). The fracture initiation pressure lies below the breakdown pressure, but is often not detectable in the pressure-time records. Therefore, $P_\mathrm {b}$ is used instead and can be interpreted as the upper limit of the fracture initiation pressure (Schmitt and Haimson 2017). The $P_\mathrm {b}$ determined in our mini-frac tests vary between 19.1 and 27.6 MPa (Fig. 5), and breakdown is well defined in all intervals.

### Instantaneous Shut-In Pressure

**Fig. 6:**
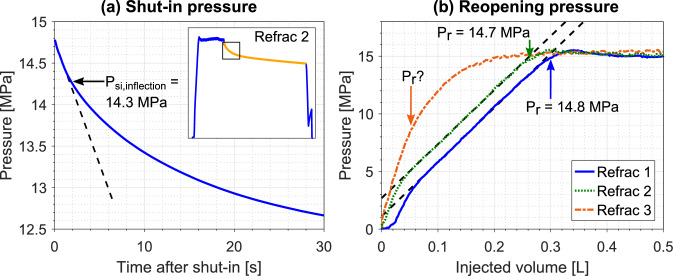
**a** Estimation of the shut-in pressure using the inflection point method ($P_\mathrm {si,inflection}$). The complete pressure recording of the second refrac cycle of the SB3.1 28 m interval is shown in the inset. The shut-in data is highlighted in orange, and the black box marks the extent of the main plot. **b** Plot of the first three refrac cycles of the same interval as in **a**. The injected volume was calculated per cycle. The fracture reopening pressure ($P_\mathrm {r}$) is picked where the pressure deviates from a linear trend, which marks a decrease in system stiffness. In the third refrac cycle, no clear reopening pressure is visible

Once pumping stops and the interval is shut-in, the pressure rapidly drops to the instantaneous shut-in pressure, hereafter referred to as shut-in pressure ($P_\mathrm {si}$). The initial pressure drop is caused by dissipation of viscous pressure losses as fluid flow and fracture growth cease. The shut-in pressure is commonly considered to be a reasonable estimate or upper bound for the minimum stress magnitude, especially for small injected fluid volumes and low viscosity fluid (Schmitt and Haimson 2017). We evaluated the shut-in pressure by two different methods. This section introduces the inflection point method (Gronseth and Kry 1981; Gronseth 1982), whereas the bilinear decay-rate method is outlined in Sect. 5.1. The shut in-pressure determined by the inflection point method ($P_\mathrm {si,inflection}$) is defined as deviation of the pressure record from a tangent line directly after pump shut-in (Fig. 6a). We used a constant time window of 30 s for all inflection point plots because the pressure transient shows only a brief phase of nearly linear pressure decline, often only 1.5–4 s long. We fitted the tangent line to this brief phase using a linear regression and visually determined when the pressure record departs from it.

**Fig. 7:**
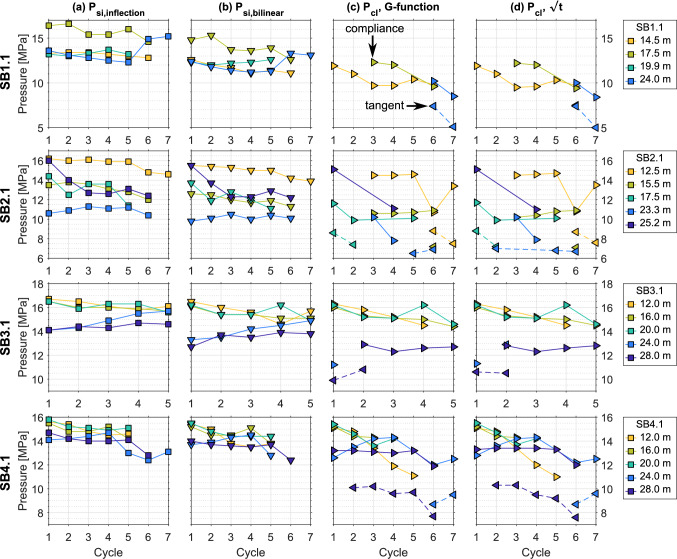
Reconciliation plots of the different mini-frac intervals for each borehole, showing variations in shut-in and closure pressure with each pressurization cycle. It should be noted that the shown values are measured surface pressures. Shut-in pressure was estimated according to the **a** inflection point method, and **b** bilinear pressure decay rate method. Tangent and compliance method picks are shown twice, **c** estimated from a G-function plot, and **d** estimated from a square root of time plot

We calculated the average $P_\mathrm {si,inflection}$ of the first five cycles and its standard deviation for each mini-frac interval. Later cycles were neglected because most of them show a larger deviation from the initial $P_\mathrm {si,inflection}$ values, and we conducted more than five cycles only in very few intervals. The obtained average $P_\mathrm {si,inflection}$ ranges from 11.1 to 16.4 MPa (Fig. 5). Before averaging the $P_\mathrm {si,inflection}$ values of the several cycles conducted at each mini-frac location, we compared them with each other. In the majority of intervals, the $P_\mathrm {si,inflection}$ values are consistent between the five to seven conducted cycles. Variations are in the range of 1–2 MPa and $P_\mathrm {si,inflection}$ rises, falls or fluctuates with advancing cycle. This is shown in so-called ‘reconciliation plots’ (Desroches and Kurkjian 1999), which were constructed for the pressure transient analysis results of all mini-frac intervals (Fig. 7a). The plots show trends of the picked pressures as the induced fracture propagates away from the borehole with each cycle. As previously mentioned, in some intervals the results of the last cycles deviate from the previous ones, particularly the cycles conducted after the step-rate injection test (usually 5th cycle). This is probably attributed to the largest amount of water being injected during the step-rate test.

We observed a ‘water hammer’ effect in the form of cyclic oscillations in the interval pressure data (for about 3 s) after pump shut-in (Fig. 4). This is likely caused by free oscillations within the hydraulic system, which arise due to the abrupt stop of the injection pump (Baumgärtner and Zoback 1989; Vermylen and Zoback 2011). The influence of this effect was minimized by applying a moving average filter (window: 0.8 s) to reveal the realistic pressure decay trend after shut-in.

### Reopening Pressure

The fracture reopening pressure ($P_\mathrm {r}$) was determined from a plot of pressure against injected volume (Fig. 6b). On such a plot, the recorded pressure during pressurization forms a straight line prior to fracture reopening, since the hydraulic system stiffness (d*P*/d*V*) is constant. The reopening of the fracture is associated with a stiffness decrease and deviation from linearity, which shows that the fracture volume connected to the hydraulic system (Baumgärtner and Zoback 1989). Figure 6b shows three successive refrac cycles with the estimated reopening pressures. A reduction of $P_\mathrm {r}$ with progressing refrac cycle is observed together with a briefer phase of linear pressure increase on a plot of pressure against injected volume. This change in the pressure data is caused by the successive increase in fracture length, which leads to a more gradual reopening of the fracture. We used only the first refrac cycle to estimate $P_\mathrm {r}$ to mitigate the effect of the gradual reopening. However, the $P_\mathrm {r}$ values of the first two to three refrac cycles usually differ less than 0.5 MPa from each other, which increases confidence in determining $P_\mathrm {r}$.

The obtained $P_\mathrm {r}$ ranges from 11.0 to 19.2 MPa (Fig. 5), which is comparable to the obtained range of $P_\mathrm {si,inflection}$. In two cases, we could not reliably determine the reopening pressure. In one case because of a potential fluid bypass around the packer and in the other case because of the development of the hydraulic fracture entirely below the packer, which both lead to reopening pressures close to the breakdown pressure. These intervals are marked as open symbols in Fig. 5.

The difference between $P_\mathrm {b}$ and $P_\mathrm {r}$ is commonly used to estimate the hydraulic tensile strength (*T*) of the rock (Bredehoeft et al. 1976; Haimson and Cornet 2003). The inferred hydraulic tensile strength lies between 5.4 and 11.6 MPa (Table 2), which is consistent with estimates from laboratory Brazilian tests (David et al. 2020). In the aforementioned intervals, where a reliable estimate of the reopening pressure was not possible, we assumed the average tensile strength in the borehole.

In most intervals, $P_\mathrm {r}$ is only slightly larger than $P_\mathrm {si,inflection}$ (< 2 MPa), which is in agreement with a compilation of a number of field test data by Sano et al. (2006) showing approximate equivalence of shut-in and reopening pressure. We need to consider that the picked reopening pressure is the apparent reopening pressure. It is larger than the true reopening pressure due to the residual permeability and gradual reopening of the hydraulic fracture (Desroches and Kurkjian 1999; Ito et al. 1999; Rutqvist et al. 2000), and the hydraulic compliance of the test equipment (Ito et al. 2006). The implications for the stress estimation will be discussed in Sect. 6.3.

### Pore Pressure

We determined the pore pressure ($P_\mathrm {p}$) from six extended overnight shut-ins (13–15 h). After the pressure inside the injection interval reached an equilibrium state, it is considered to be a good approximation for the local pore pressure. Before conducting the mini-frac tests, the boreholes had a low outflow rate that was not measured but was approximately in the order of a few liters per hour. This indicates that some natural fractures intersecting the borehole are permeable (see Sect. 4.5), as the Rotondo granite only has a permeability in the order of $10^{-18}\,\hbox {m}^{2}$ (David et al. 2020). We assumed that the hydraulic fractures created during the mini-frac tests likely connect to one of these permeable structures, which will be discussed in Sect. 6.2. Therefore, it is possible to equilibrate the interval with the surrounding rock mass on the time scale studied.

**Fig. 8:**
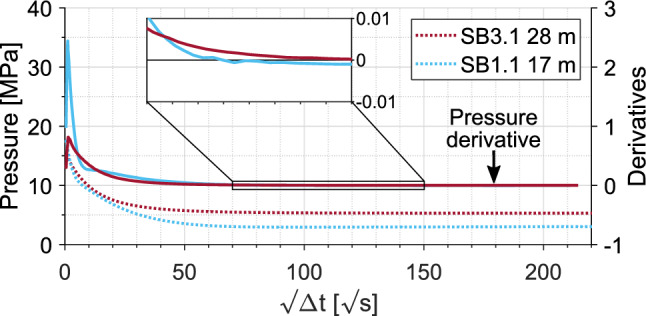
Two examples of an overnight shut-in, showing the pressure transient (dotted line) and its derivative (solid line). Displayed are the last refrac cycle (SB1.1) and initial frac cycle (SB3.1), which lasted 13 h and 13.8 h, respectively. We estimated the local pore pressure from the pressure curve once the derivative reached a value close to zero, which is detailed in the inset. Note that the shown values are measured surface pressures (colour figure online)

The measured pore pressures lie between 2.0 and 5.6 MPa (Fig. 5), and generally an increase in pore pressure with depth is observed. The hydrostatic pore pressure at the top of the four boreholes would be between 9.2 and 10.9 MPa, taking into account the varying overburden. Figure 8 shows two examples of overnight shut-ins. To compress the time scale, the data are plotted against the square root of time. The pressure derivative stabilizes close to zero after about 2.8 h (equals ${100}\,{\sqrt{\mathrm{s}}}$) and 4.7 h (equals ${130}\,{\sqrt{\mathrm{s}}}$) to a pressure of 3.2 MPa and 5.6 MPa for SB1.1 and SB3.1, respectively. In SB1.1, the interval pressure is slightly increasing at late time, seen by the negative derivative. This is possibly caused by inflow from conductive natural fractures into the hydraulic fracture and borehole.

### Hydraulic Fracture Orientation and Natural Fractures

**Fig. 9:**
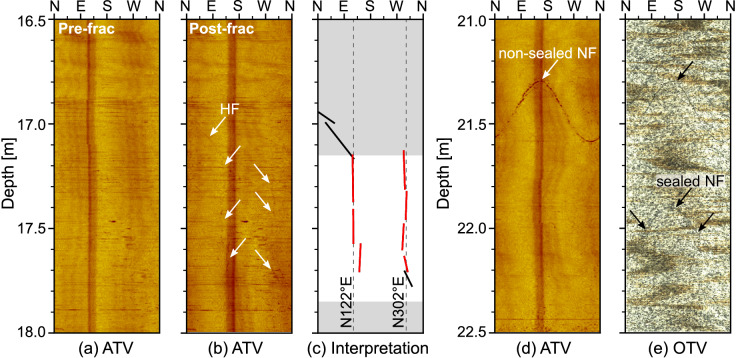
Examples of amplitude images from unwrapped acoustic televiewer logs **a** before and **b** after the hydraulic fracturing test at 17.5 m depth in SB1.1. **c** Interpreted subvertical hydraulic fracture (HF), where the most vertical parts (in red) were used to calculate the mean strike direction, indicated by dashed lines. The gray shaded areas show the packer locations. In addition, an example of a non-sealed natural fracture (NF) and sealed NF are shown in an unwrapped **d** acoustic televiewer and **e** optical televiewer log (colour figure online)

We identified the created hydraulic fractures on acoustic televiewer logs (ATV), and they are vertical or steeply dipping ($>{80}^{\circ }$) in most intervals. The creation of longitudinal fractures at the borehole wall supports the assumption that the overburden stress is larger than at least one of the horizontal stresses and approximates a principal stress direction. The possibility that the fractures rotate into a horizontal orientation away from the borehole is very unlikely, as thrust faulting can most probably be ruled out in the study area. Figure 9a–c shows an example of ATV amplitude images of a vertical hydraulic fracture in borehole SB1.1 and its interpreted orientation. As can be seen in Fig. 9c, the hydraulic fracture extended below the contact zone of packer and borehole wall (visualized as gray shaded area), which was observed in almost all intervals. Based on the vertical, $>{80}^{\circ }$ dipping parts of the interpreted hydraulic fracture, we evaluated that the average direction of the maximum horizontal stress ($S_\mathrm {Hmax}$) is approximately $\hbox {N}\,{100}^{\circ }-{120}^{\circ }\hbox {E}$. The observed hydraulic fractures in SB2.1 are an exception, since they dip between ${65}^{\circ }$ and ${80}^{\circ }$. The possibility that these intervals contain reactivated natural fractures or that the stress state is rotated, e.g. due to the enlarged niche of the BedrettoLab, cannot be ruled out and needs further investigation.

Figure 9d, e shows how we differentiated between sealed and non-sealed natural fractures. A fracture was identified as non-sealed, i.e. having a significant aperture at the borehole wall, if it was visible on both ATV and OTV logs. On the contrary, if a fracture was only visible on the OTV log, we identified it as sealed. All four boreholes studied contain both types of fractures, but there are also longer sections of intact rock, i.e. without any pre-existing natural fractures.

## Elaborated Pressure Transient Analysis

Having presented the basic data analysis of the mini-frac tests, we move on to a more detailed and elaborated analysis of the pressure transient data. As mentioned in the introduction, either the instantaneous shut-in pressure ($P_\mathrm {si}$) or the fracture closure pressure ($P_\mathrm {cl}$) needs to be determined to estimate the magnitude of the least principal stress ($S_\mathrm {3}$). Different definitions for $P_\mathrm {cl}$ exist in the literature. We follow the definition of McClure et al. (2014, 2016) that defines closure pressure as the pressure at which a fracture mechanically starts to close, which means that the fracture walls come into contact. Hence, $P_\mathrm {cl}$ is nearly equal to but just less than the magnitude of $S_\mathrm {3}$, which acts onto the fracture walls. Consequently, $P_\mathrm {cl}$ is the better estimate of the $S_\mathrm {3}$ magnitude, but for the small injection volume of mini-frac tests, it is sometimes difficult to actually distinguish $P_\mathrm {si}$ and $P_\mathrm {cl}$ (Schmitt and Haimson 2017).

Several methods and diagnostic plots have been proposed to estimate these pressures from mini-frac tests. In the following, we compare five common methods to estimate the magnitude of $S_\mathrm {3}$ (Table 1): (1) shut-in pressure from the inflection point method as introduced in Sect. 4.2 (Gronseth and Kry 1981; Gronseth 1982), (2) shut-in pressure from the bilinear pressure decay rate method (Tunbridge 1989; Lee and Haimson 1989), (3) fracture closure pressure picked by the tangent method (Barree et al. 2007), (4) fracture closure pressure picked by the fracture compliance method (McClure et al. 2014, 2016; Jung et al. 2016), and (5) jacking pressure from the step-rate injection tests (Doe and Korbin 1987; Rutqvist and Stephansson 1996).

All methods have in common that they look for a point during the shut-in period that reflects a change in leak-off behavior. The measured pressure transient curves show great variety between the boreholes, between the different depth intervals and between cycles inside the intervals itself. Because the shut-in time varies between cycles and intervals, the total pressure drop cannot be compared. Most cycles typically show a convex pressure transient and the pressure drops in the order of 5–10 MPa in roughly about 3 min. Some transient curves have smaller pressure drops about 1–4 MPa which is typically associated with flatter and less convex shape (for example in Fig. 3).

In the next sections, we will introduce the five methods mentioned above for estimating the magnitude of $S_\mathrm {3}$, followed by examples of their application and a summary comparison of all methods. The first method to estimating the magnitude of $S_\mathrm {3}$, the inflection point method ($P_\mathrm {si,inflection}$), was already described in Sect. 4.2.

### Bilinear Pressure Decay Rate Method

**Fig. 10:**
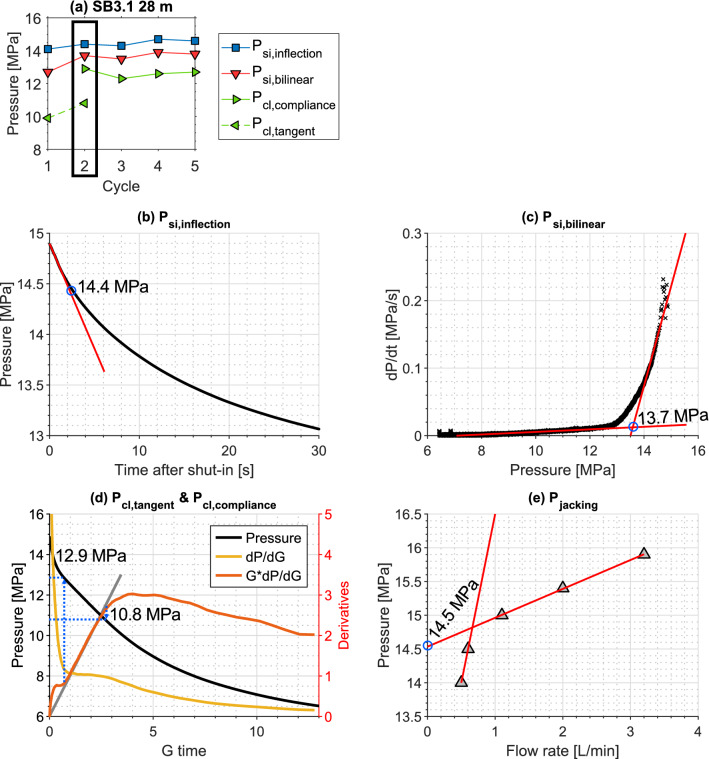
Plots of the different method to estimate $S_\mathrm {3}$ for the first refrac cycle (SB3.1 28 m). The shut-in duration was 60 min. **a** Reconciliation plot showing the results of the four methods presented in **b**–**d**. The rectangle marks the displayed cycle. **b** Inflection point method, **c** bilinear pressure decay rate method, **d** tangent and fracture compliance method, **e** jacking pressure from step-rate injection (last cycle, shown in Fig. 12)

In addition to the inflection point method, we used the bilinear pressure decay rate method to estimate the shut-in pressure. Two linear regressions were fitted to the data on plots of pressure derivative against injection pressure (d*P*/d*t* vs. *P*) (Tunbridge 1989; Lee and Haimson 1989): The first regression approximates the strong decrease of d*P*/d*t* shortly after shut-in, where it does not deviate too far from a straight line. The second regression approximates the derivative where it approaches a nearly constant value close to a horizontal zero line. The intersection of the two regressions gives the shut-in pressure ($P_\mathrm {si,bilinear}$). The bilinear pressure decay rate method is presented in Fig. 10c. We noticed that especially the linear fit to the strong decrease in pressure derivative is ambiguous. Thus, we always fitted the data after the first second or two, during which data points are widely spaced and potentially biased by the moving average filter, until the end of a linear trend.

### G-Function and Fracture Closure Methods

**Fig. 11:**
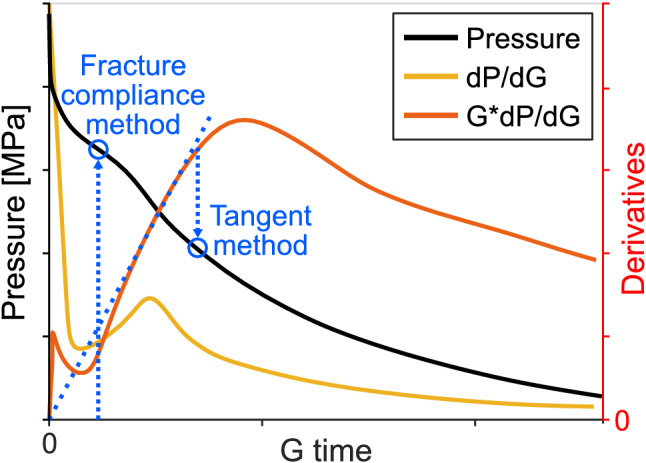
Comparison of the two methods that estimate fracture closure pressure on a G-function plot: the tangent method and the fracture compliance method. A hypothetical pressure decline curve directly after shut-in is shown

In contrast to the two shut-in pressure methods ($P_\mathrm {si,inflection}$ and $P_\mathrm {si,bilinear}$), the two methods to determine the fracture closure pressure ($P_\mathrm {cl}$) utilize the so-called G-function. The G-function is a specialized time-dependent function relating shut-in time to injection duration (Nolte 1979), so that under certain assumptions the G-function is linearly proportional to the cumulative volume of fluid leaked off from the fracture after shut-in (see Appendix 1). A G-function plot usually includes the pressure, its derivative, d*P*/d*G*, and its semi-logarithmic derivative (Bourdet et al. 1989), G $\cdot$ d*P*/dG (see Appendix 2). Alternatively, a pressure versus square root of time plot can be used to determine the fracture closure pressure. Our analysis showed that the results between the two different plots agree in most cases (Fig. 7c+d). We therefore combined the results of both plots to calculate the mean closure pressure and its standard deviation, but show only the G-function graphs hereafter. As mentioned above, two commonly used methods exist to determine the fracture closure pressure, the tangent and fracture compliance method. The tangent method, following the terminology of Jung et al. (2016), is a widely used method in petroleum engineering and was introduced by Barree et al. (2007). They showed that drawing a straight line through the origin and tangential to the semi-logarithmic derivative on a G-function plot (G $\cdot$ d*P*/dG) yields $P_\mathrm {cl,tangent}$ at the departure or down curvature from this straight line. Figure 11 shows a hypothetical G-function plot and the application of the tangent method. Most of the obtained semi-logarithmic derivatives of the pressure transients deviate from the linear trend ideally starting at the origin, as illustrated by the small peak at early time in Fig. 11. Several, partly contradictory, interpretations exist for the trends in the semi-logarithmic derivative [see Jung et al. (2016) for a review], e.g. the gradual closure of the hydraulic fracture associated with increasing fracture stiffness, near-borehole complexity, or multiple closures of intersected natural fractures (see Sect. 6.2).

Because the tangent method may significantly underestimate $S_\mathrm {3}$ in low permeability formations (McClure et al. 2014, 2016; Wang and Sharma 2017, 2019), an alternative method to pick the onset of fracture closure was proposed by McClure et al. (2014, 2016). The method is referred to as fracture compliance method, and the closure pressure ($P_\mathrm {cl,compliance}$) is picked when the semi-logarithmic derivative begins to curve upward, which is caused by an increase in fracture stiffness (or decrease of fracture compliance, which is the inverse of stiffness). This increase in fracture stiffness is caused by the gradual fracture closure from tip to borehole, and has a dominant effect on the pressure transient (McClure et al. 2014, 2016; Jung et al. 2016; Wang and Sharma 2019; see also Appendix 1). Figure 11 illustrates the application of the compliance method and shows that the determination of the point where the semi-logarithmic derivative curves upward is subjective.

### Jacking Pressure

**Fig. 12:**
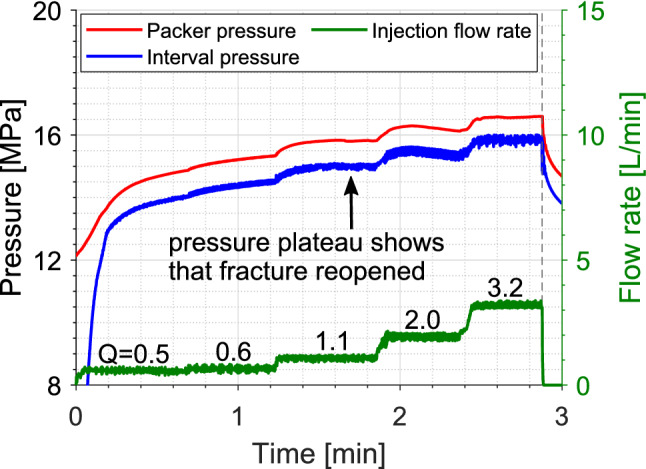
Step-rate injection test with five pressurization steps in SB3.1 (28 m). During the initial two pressurization steps, the interval pressure is rising, which is interpreted as flow into the rock matrix and a closed fracture. Starting with the third pressurization step, the pressure plateaus, which is associated with an opening of the hydraulic fracture. The pressure and flow rate values are plotted in Fig. 10e

Another estimate of the $S_\mathrm {3}$ magnitude is given by the jacking pressure ($P_\mathrm {jacking}$) from the step-rate injection tests (Doe and Korbin 1987). Since different methods to determine $P_\mathrm {jacking}$ exist in the literature, we followed the approach of Rutqvist and Stephansson (1996). Similarly to other approaches, two linear regression can be fitted to the data points on a pressure versus flow rate plot (*P* vs. *Q*). The bilinear pattern is associated with flow into the rock matrix before the fracture has opened and flow into the open fracture once the pressure is high enough (Fig. 12). $P_\mathrm {jacking}$ is picked as backward extrapolation of the second linear regression to a flow rate of $Q={0}\text { L min}^{-1}$ (Fig. 10e). During the experiments, we found that the hydraulic fracture often reopens during the first pressurization step, because already low flow rates generate sufficiently high pressures in the low-permeable granite. The chosen method nevertheless allows a determination of the jacking pressure, since only the linear regression associated with the flow into the open fracture is needed.

### Results of Two Example Intervals

**Fig. 13:**
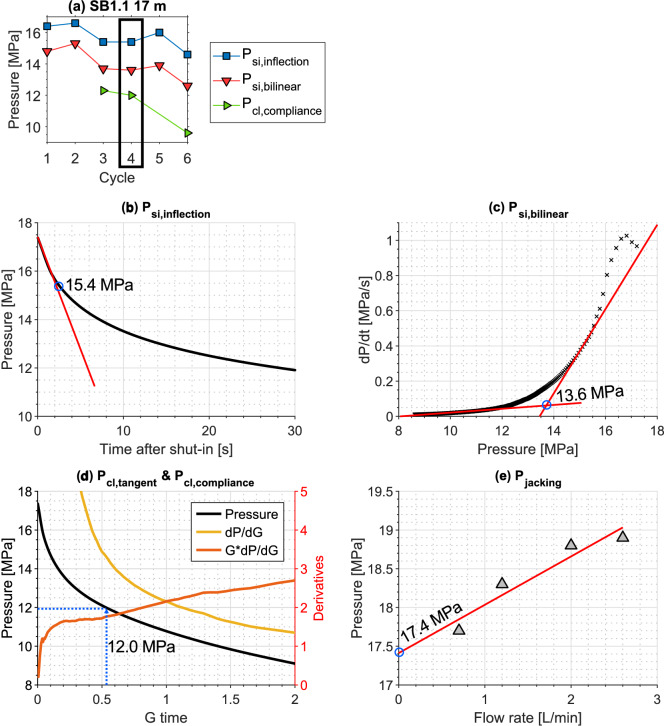
Plots of the different method to estimate the $S_\mathrm {3}$ magnitude for the third refrac cycle (SB1.1 17.5 m). The shut-in duration was 3.3 min. **a** Reconciliation plot showing the results of the three methods presented in **b**–**d**. The rectangle marks the displayed cycle. **b** Inflection point method, **c** bilinear pressure decay rate method, **d** fracture compliance method (tangent method not applicable), **e** jacking pressure from step-rate injection (second last cycle)

As an illustration, we present two example intervals to compare the aforementioned five methods. The first example interval is located in SB3.1 at 28 m. Figure 10 shows the plots associated with the five methods and their estimates of the $S_\mathrm {3}$ magnitude. All methods except the jacking pressure are shown for the first refrac cycle, which has an extended shut-in time of 1 h. A second example interval is shown in Fig. 13 (SB1.1 at 17.5 m), to illustrate the effects of the standard shut-in time of about 3 min. In the next paragraphs, we will present the first example (Fig. 10) in detail.

The inflection point method leads to a $P_\mathrm {si,inflection}$ value of 14.4 MPa in the example interval (Fig. 10b). The reconciliation plot in Fig. 10a shows the results of all five cycles. The $P_\mathrm {si,inflection}$ values are fairly consistent between cycles and vary within a range of 0.6 MPa.

The bilinear pressure decay rate method yields a slightly lower shut-in pressure ($P_\mathrm {si,bilinear}$) of 13.7 MPa (Fig. 10c). Two linear trends and the corresponding linear regressions can be seen on the d*P*/d*t* vs. *P* plot, one approximating the largest decrease in d*P*/d*t* and the other one close to a horizontal zero line. Early time noise at high pressures was neglected for the calculation of the linear regression. The reconciliation plot (Fig. 10a) shows that $P_\mathrm {si,bilinear}$ is on average 1 MPa lower than $P_\mathrm {si,inflection}$. In general, they follow the same trend, but $P_\mathrm {si,bilinear}$ shows a slightly higher variation of 1.2 MPa over all five cycles.

Figure 10d shows the G-function plot of the example interval, which was used to determine the fracture closure pressure with the tangent and compliance method. The semi-logarithmic derivative, G $\cdot$ d*P*/dG (orange line in Fig. 10d), exhibits a distinct maximum to which the tangent method can be applied. The deviation from the straight line, starting at the origin, results in a $P_\mathrm {cl,tangent}$ value of 10.8 MPa. On the contrary, the compliance method picks fracture closure at the point where the semi-logarithmic derivative increases after its plateau at a G time of 0.5. This yields a $P_\mathrm {cl,compliance}$ value of 12.9 MPa. Both methods, the tangent and compliance method, always provide stress estimates below those of the two shut-in pressure methods. In addition, $P_\mathrm {cl,tangent}$ is consistently lower than $P_\mathrm {cl,compliance}$.

**Fig. 14:**
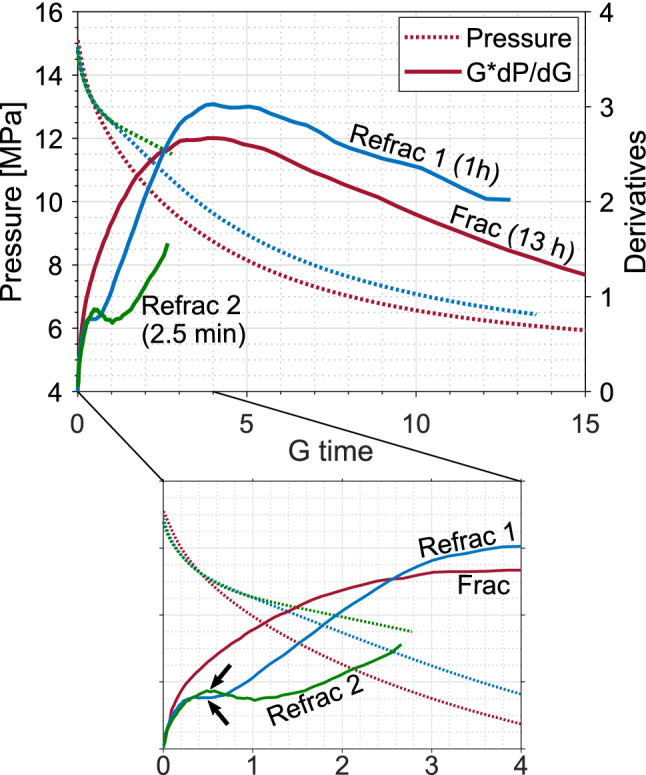
G-function plot of the pressure transient and semi-logarithmic derivative for the frac (red lines), first refrac (blue lines, also shown in Fig. 10) and second refrac cycle (green lines) of the SB3.1 28 m interval. The shut-in observation times are given in brackets. The enlarged plot and arrows highlight the hump (refrac 2) and plateau (refrac 1) at early time. Both features do not occur in the frac cycle. Refrac 2 does not reach the global maximum of the semi-logarithmic derivative, which prevents the use of the tangent method

The observed shapes of the pressure and semi-logarithmic derivatives versus G time varied significantly between cycles and intervals. In the example interval, the compliance method was successfully applied in all cycles except the frac cycle. Here it could not be applied because the semi-logarithmic derivative has the form of an ‘ideal’ pressure transient curve (Jung et al. 2016), meaning that it is linear until reaching the peak and then curves downward and monotonically decreases (frac cycle in Fig. 14). However, the following cycles, and many cycles in general, show a pronounced down deflection of the semi-logarithmic derivative at early time (e.g., first and second refrac cycle in Fig. 14). In some cases, the semi-logarithmic derivative exhibits an early local maximum with varying amplitude; in other cases, it plateaus. The fracture compliance method was always applied after this plateau or the local minimum following the local maximum. The feature is interpreted as the closure of pre-existing natural fractures, which occur before or simultaneously with the closure of the hydraulic fracture. This is discussed in more detail in Sect. 6.2.

In contrast to the compliance method, the tangent method could only be applied to the frac and first refrac cycle in the example interval, both of which have extended shut-in times. All other cycles had too short shut-in times below 3 min and the minimum shut-in time needed to apply the tangent method is, with a few exceptions, more than 15 min. Thus, most short shut-ins give an increasing semi-logarithmic derivative at the end of the observation time and the feature picked by the tangent method comes up after the time series, which is seen, for example, in Fig. 13d. Only extended shut-in times reach a late time monotonic decline (Fig. 14).

The last method is the jacking pressure, which was estimated from the step-rate injection test of each interval. Figure 10e shows the pressure versus flow rate plot of this step-rate injection (injection protocol shown in Fig. 12), which is cycle five in the example interval. Two distinct linear trends can be seen, where the last three flow rates correspond to flow into an open fracture. The extrapolation of this second linear trend yields a $P_\mathrm {jacking}$ value of 14.4 MPa. Since in most intervals the lowest flow rate already reopened the hydraulic fracture, most step-rate tests were conducted with only three to four flow rate steps, such as shown in Fig. 13e.

**Fig. 15:**
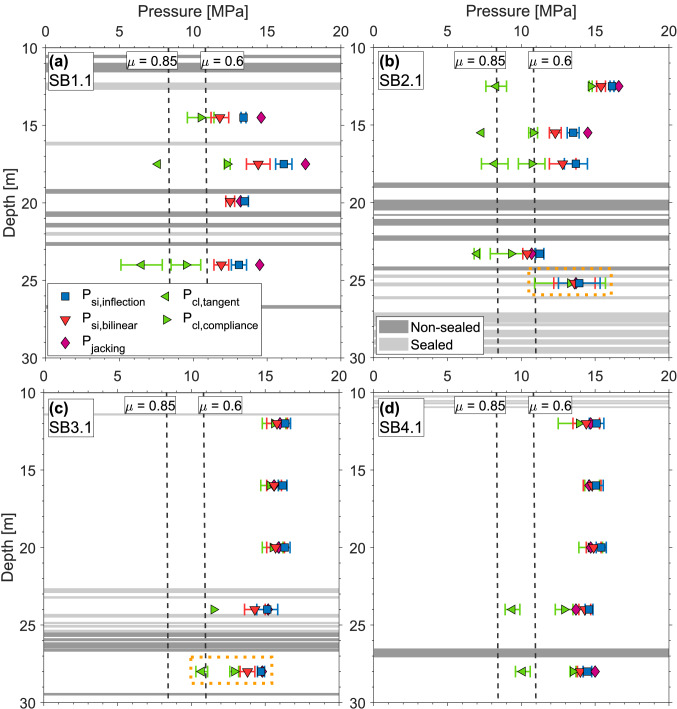
Comparison of the five different methods to estimate $S_\mathrm {3}$ for each of the four vertical boreholes (values given in Table 2). For each interval, the mean and its standard deviation are displayed (see Fig. 7 for individual values). The two dashed lines show the lower bound of $S_\mathrm {3}$ according to the frictional limit calculated by Eq. (1). Shaded areas visualize the depth where natural fractures were identified at the borehole wall on optical and acoustic televiewer logs. The two example intervals (Figs. 10, 13) are indicated by boxes

For our example interval in SB3.1, we obtained the following mean values and standard deviations, which are visualized among the other SB3.1 intervals in Fig. 15c: the highest $S_\mathrm {3}$ estimates are given by $P_\mathrm {jacking}$ and $P_\mathrm {si,inflection}$, with 14.8 MPa and $14.7 \pm 0.3$ MPa, respectively. These are followed by $P_\mathrm {si,bilinear}$ with $13.8 \pm 0.5$ MPa and $P_\mathrm {cl,compliance}$ with $12.9 \pm 0.3$ MPa. The lowest estimate is $P_\mathrm {cl,tangent}$ with $10.7 \pm 0.4$ MPa.

### Compilation of Results and Comparison of Methods

The mean $S_\mathrm {3}$ estimate of each method and its standard deviation were calculated for the 19 mini-frac intervals. The results are listed in Table 2 and displayed in Fig. 15. No measure of variation can be determined for the jacking pressure because we only performed one step-rate test per interval. Most step-rate tests were conducted with only three or four different flow rates, thus its uncertainty must be considered when interpreting the jacking pressure. Figure 7 shows a combination of the reconciliation plots of all intervals of each borehole. Any outliers, which were identified on the previously introduced reconciliation plots, were excluded in the averaging process.

In brief, we calculated the following overall mean $S_\mathrm {3}$ estimates by averaging over all intervals: the mean $P_\mathrm {si,inflection}$ and $P_\mathrm {jacking}$ are nearly identical, with $14.6 \pm 1.4$ MPa and 14.8 ± 1.5 MPa, respectively. Slightly lower is the $P_\mathrm {si,bilinear}$ mean (13.8 ± 1.6 MPa) and the $P_\mathrm {cl,compliance}$ mean ($12.9 \pm 2.1$ MPa). The $P_\mathrm {cl,tangent}$ mean is the lowest with 8.3 ± 1.5 MPa.

Our results indicate that the different methods to estimate $S_\mathrm {3}$ can be ranked as follows: $P_\mathrm {cl,tangent}$ gives the smallest magnitude, followed by $P_\mathrm {cl,compliance}$, $P_\mathrm {si,bilinear}$ and $P_\mathrm {si,inflection}$. $P_\mathrm {jacking}$ lies close to $P_\mathrm {si,inflection}$ and is smaller in some intervals and larger in others. The difference between $P_\mathrm {si,inflection}$ and $P_\mathrm {jacking}$ is less than 1.5 MPa and between $P_\mathrm {si,inflection}$ and $P_\mathrm {si,bilinear}$ less than 1.7 MPa. The $P_\mathrm {r}$ values are larger than most estimates of $S_\mathrm {3}$, but in a few exceptions $P_\mathrm {si,inflection}$ and $P_\mathrm {jacking}$ are higher.

The two shut-in pressure methods, inflection point and bilinear decay rate, are very robust and were applied to nearly all cycles. Their picks are relatively early after shut-in, giving high pressure values. Also, the jacking pressure was estimated from all except one step-rate tests. The problematic test at 24 m in SB4.1 yielded lower pressures with increasing flow rates, possibly because of fracture propagation and interaction with natural fractures. On the opposite, the fracture closure pressure determined on G-function plots is picked later and always yields lower pressures. The tangent method was applicable only in 9 of the 19 intervals. In Table 2 a missing standard deviation indicates intervals where the tangent pick was possible only for a single cycle.

## Discussion

### Comparison of Methods to Determine the Magnitude of $S_\mathrm {3}$

Since the different methods to estimate the $S_\mathrm {3}$ magnitude yield discrepancies of up to 10 MPa, we discuss their results in ascending order. The tangent method gives by far the lowest stress estimates; hence, we first compared all estimates against the theoretical lower bound of $S_\mathrm {3}$ assuming that the rock mass contains critically stressed faults that limit its strength (Townend and Zoback 2000; Zoback and Townend 2001). This frictional limit recognizes that the maximum ratio of the maximum and minimum effective stresses is limited by the faults and fractures present in a rock mass. Figure 15 visualizes that a number of pre-existing natural fractures intersect all boreholes. We analyzed their orientation relative to our estimated $S_\mathrm {Hmax}$ direction ($\hbox {N}\,{100}^{\circ }-{120}^{\circ }\hbox {E}$). In each borehole, several fractures are oriented favorably for reactivation, meaning they are striking E–W or SE–NW and are steeply dipping ($>{60}^{\circ }$). This justifies the use of the frictional limit. In the context of a normal faulting (and transitional strike-slip) stress regime, i.e. that the vertical stress ($S_\mathrm {v}$) is the maximum principal stress, the limiting $S_\mathrm {3}$ magnitude is calculated according to:

1 $$\begin{aligned} S_\mathrm {3} \ge \frac{S_\mathrm {1}-P_\mathrm {p}}{[(\mu ^2+1)^{1/2}+\mu ]^2}+P_\mathrm {p} \end{aligned}$$

For the pore pressure $P_\mathrm {p}$, we use the mean value across all boreholes, which is 3.3 MPa. The resulting $S_\mathrm {hmin}$ limits for a location 30 m below the BedrettoLab, which corresponds to the maximal depth of the SB boreholes, are 8.4 MPa and 10.9 MPa, assuming a frictional coefficient $\mu$ of 0.6 and 0.85, respectively. Figure 15 shows the two frictional limit lines next to the $S_\mathrm {3}$ estimates of the different methods. It should be noted that this is just a hypothetical lower limit, which is strongly influenced by the input parameters used, such as $P_\mathrm {p}$ and $\mu$. For example, if we used a higher $P_\mathrm {p}$ value in Eq. (1), we would obtain a higher $S_\mathrm {3}$ limit. The tangent method estimates stress magnitudes as low as 6.2 MPa, which is clearly below the permitted value in situ under the assumptions on friction and pore pressure used. Therefore, the estimation by the tangent method is of questionable application to our data set.

The fracture compliance method gives stress estimates that are above the frictional limit for $\mu = 0.85$ in all intervals and above the limit for $\mu = 0.6$ in most intervals. Further, its estimates are close (<1 MPa) to the estimate of at least one of the two shut-in pressure methods in more than half of the intervals. Nevertheless, the compliance method gives the second lowest stress estimate in all intervals because fracture closure is picked later and at lower pressures than the shut-in pressure. The compliance method was first applied to larger scale hydraulic fracturing operations in the unconventional petroleum industry, i.e. in low-permeability reservoirs. It is debatable whether fracture closure can be picked for small-scale mini-frac tests in a crystalline rock mass. Several authors have argued that it is likely that small scale hydraulic fractures in vertical wells and low-permeability formations close immediately once pumping has stopped (Zoback and Kohli 2019; McClure et al. 2019). This rapid closure of the hydraulic fracture after shut-in can explain that most of the stress estimation methods yield comparable values within a narrow range. Such rapid closures can occur because of the low volume of injected fluid or the intersection of highly permeable natural fractures. Dutler et al. (2020) and McClure et al. (2019) stated that the intersection of highly permeable natural fractures leads to monotonically declining plots of d*P*/dG against the G-function. McClure et al. (2019) interpreted this monotonically declining derivative as the instant contact of the fracture walls after shut-in and suggested that in this case the shut-in pressure can be taken as an estimate of $S_\mathrm {3}$. We observed a monotonically declining pressure derivative on G-function plots in most of our data (e.g., Fig. 13d), and only in a few intervals and cycles the pressure derivative exhibits a distinguishable minimum and maximum or a small plateau (e.g., Fig. 10d). This suggests that the hydraulic fractures in our mini-frac tests exhibit a rapid closure in almost all cycles. This rapid closure and the variability in the pressure derivatives on G-function plots justify that the compliance method gives a reasonable $S_\mathrm {3}$ estimate in most intervals, although it involves more processing than the shut-in pressure methods.

Compared to the two methods of estimating fracture closure, the two shut-in pressure methods are more straightforward to apply and yield similar magnitudes of $S_\mathrm {3}$. The bilinear decay rate method yields stress magnitudes that are on average 0.8 MPa lower than magnitudes from the inflection point method. Notably, this difference between the shut-in pressure methods, and the scatter of all methods in general, is the largest in the shallow intervals of SB1.1 and SB2.1, but in SB3.1 and SB4.1 it is larger in the deep intervals. Possibly this arises from the complex interaction with natural fractures. The two shut-in pressure methods are applicable in nearly every cycle. The derived standard deviation indicates the $S_\mathrm {3}$ variability and uncertainty in each interval. Comparing the two methods, others have argued that the bilinear decay method is less subjective than the graphical inflection point method (Guo et al. 1993b; Chang et al. 2014). Certainly, the inflection method depends on the time window the picking is done, so we used a consistent window of 30 s. We consider that both shut-in pressure methods can be used to estimate $S_\mathrm {3}$, and we use the results of the inflection point method in the stress analysis in Sect. 6.3.

The step-rate tests yield a jacking pressure close to the two shut-in methods, which is similar to other studies (e.g. Doe and Korbin 1987; Dutler et al. 2020). A disadvantage of the jacking pressure is that in our data set it gives only a single stress estimate per interval. Nevertheless, it is useful to determine the jacking pressure, as its proximity to the shut-in pressure values increases the confidence in the estimated stress magnitude.

Dutler et al. (2020) investigated how to determine $S_\mathrm {3}$ from six hydraulic fracturing experiments in granodiorite at the Grimsel Test Site. They supported their results by estimating fracture closure using measured displacements from an array of strain gauges. Their findings are comparable to ours: the stress estimates of the two shut-in pressure methods and the compliance method fall into a narrow range, but the tangent method underestimates the stress magnitude. Moreover, they suggested that the hydraulic fractures experience a rapid closure because of the intersection with highly permeable, natural fractures. The authors recommended using shut-in pressure methods for estimating the stress magnitude rather than the closure pressure (tangent or compliance) methods. This supports our findings, although the injected volume at the Grimsel Test Site was considerably larger than during our mini-frac tests.

### Interaction of Hydraulic and Natural Fractures

Apart from the closure process of the hydraulic fracture, the intersection and closure of pre-existing natural fractures has an effect on the shape of the pressure transient. As already stated in the results, many plots of the semi-logarithmic derivatives versus G time show a distinct small peak or plateau at early time, which we interpret as the closure signal of natural fractures. For example, Fig. 14 shows a case where the semi-logarithmic derivative of the initial frac cycle has only a single large peak and the first and second refrac cycle show a clear small peak at early time. Generally, it is often the first cycle(s) that lack the down deflection or peak at early time. A possible explanation is that the intersection of the hydraulic fracture with one or more natural fractures occurs during one of the intermediate refrac cycles and not before (Dutler et al. 2020). This is more likely as the hydraulic fracture propagates further away from the borehole with additional cycles. Other studies have hypothesized that this interaction of the hydraulic fracture with natural fractures or weakness zones can explain observed orientation changes of the microseismic cloud during successive refrac cycles of hydraulic simulations (Gischig et al. 2018; Dutler et al. 2020).

The clear closure signals of intersected natural fractures on the G-function plots have been observed in numerous other field studies and numerical models (Wallace et al. 2014; Jung et al. 2016; Nadimi et al. 2020; Kamali and Ghassemi 2019; Wang and Sharma 2019). The interaction of the propagating hydraulic fracture with differently oriented natural fractures will cause changes in the pressure transient, as the closure of reactivated natural fractures will change the system stiffness/compliance and, if hydraulic connectivity is lost, the leak-off surface area. Many factors influence the closure process itself, e.g., the intersection angle, the horizontal differential stress, and the rock, natural fracture and fracturing fluid properties (Blanton 1982; Warpinski and Teufel 1987; Zhou et al. 2008).

Kamali and Ghassemi (2019) showed in their simulations how the slope of the pressure transient and signatures on G-function plots depend on the intersection angle and the fracture stiffness of the natural and hydraulic fracture itself. Their simulations produced similar features in the semi-logarithmic derivative on G-function plots as we observe in our data, namely, the down deflections and small peaks at early time. An important finding of their study is that the closure of the whole fracture system is dependent on the fracture stiffness contrasts and intersection angles. It is possible that the closure signature of the hydraulic fracture is masked by or combined with the closure signature of one or multiple natural fractures. This makes the interpretation of closure pressure challenging, especially when using the compliance method.

### Stress Field Around the BedrettoLab

**Fig. 16:**
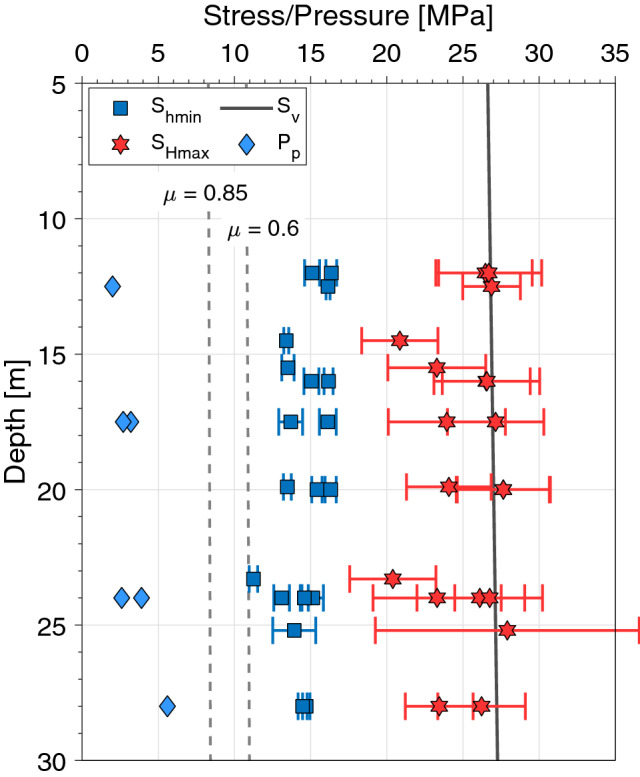
In situ stress magnitudes and pore pressures from 19 mini-frac tests distributed over four vertical boreholes. The vertical stress ($S_\mathrm {v}$) was calculated by integrating the overburden density. The minimum horizontal stress ($S_\mathrm {hmin}$) was determined by the inflection point method, and the maximum horizontal stress ($S_\mathrm {Hmax}$) was calculated using Eq. (3). Error bars are from the standard deviation. The two dashed lines depict the lower bound of $S_\mathrm {3}$ according to the frictional limit (Eq. 1) using the mean pore pressure of 3.3 MPa. The $S_\mathrm {Hmax}$ estimates are in the range of the $S_\mathrm {v}$ magnitude, suggesting a stress state that is transitional between normal and strike-slip faulting

Following the previous discussion, we favor the shut-in pressure from the inflection point method as a reasonable estimate of $S_\mathrm {3}$. Its standard deviation per interval is mostly below 0.8 MPa. Overall, the $S_\mathrm {3}$ stress magnitude ranges between 11.2 and 16.4 MPa with a mean value of $14.6 \pm 1.4$ MPa (Fig. 16).

As mentioned in the site description, thrust faulting can be excluded as the local stress regime (Gischig et al. 2020; Ma et al. 2020). In the following, we assume that topographic influences are minor, that one principal stress component is vertical ($S_\mathrm {v}$), and that the other two principal stress components lie in a horizontal plane ($S_\mathrm {hmin}$, $S_\mathrm {Hmax}$). Since we expect the stress regime to be either normal or strike-slip faulting, the maximum horizontal stress $S_\mathrm {Hmax}$ corresponds either to the intermediate principal stress $S_\mathrm {2}$ (normal faulting) or to the major principal stress $S_\mathrm {1}$ (strike-slip).

The magnitude and orientations of the two horizontal stresses can be inferred from mini-frac tests. Unlike the minimum horizontal stress $S_\mathrm {hmin}$ magnitude, the $S_\mathrm {Hmax}$ magnitude cannot be directly inferred from the pressure transient curve. However, the cylindrical borehole introduces a stress perturbation in the rock mass, leading to a stress concentration at the borehole wall. This is described by the classical Kirsch equations. Based on these equations, the criterion for fracture initiation in an initially impermeable, homogeneous, isotropic, linearly elastic medium is (Hubbert and Willis 1957; Scheidegger 1960):

2 $$\begin{aligned} P_\mathrm {b} = 3 \cdot S_\mathrm {hmin} - S_\mathrm {Hmax} + T - P_\mathrm {p}. \end{aligned}$$

To estimate $S_\mathrm {Hmax}$, we made two additional assumptions. First, the unknown hydraulic tensile strength of the rock *T* was replaced by the difference between the breakdown and reopening pressure ($P_\mathrm {b} - P_\mathrm {r}$) in Eq. (2) (Bredehoeft et al. 1976), which yields Eq. (3). Hereby, we assume that the fracture fully closes after the frac cycle. This assumption is commonly made and substitutes laboratory measurements on core samples to measure the rock’s tensile strength (e.g. Haimson and Cornet 2003; Wang et al. 2017). Second, we used the pore pressures $P_\mathrm {p}$ inferred from overnight shut-ins for the respective intervals, and in intervals without overnight shut-in we used the average borehole estimate.

3 $$\begin{aligned} S_\mathrm {Hmax} = 3 \cdot S_\mathrm {hmin} - P_\mathrm {p} - P_\mathrm {r}. \end{aligned}$$

Equations (2) and (3) are both ignoring poroelastic effects and incorporate Terzaghi’s effective stress law. Several other breakdown models were developed beyond the Hubbert–Willis breakdown model (Eq. 2) and take into account effects of poroelasticity or fluid penetration into the rock matrix prior to fracture initiation (e.g. Rummel 1987; Schmitt and Zoback 1989, 1993). We did not incorporate more sophisticated models, because the underlying formulas require several laboratory measurements to determine parameters like the Biot poroelastic parameter or rock elastic moduli.

Many studies have addressed other factors besides poroelasticity that affect the reliability of the $S_\mathrm {Hmax}$ estimation using Eq. (3) (e.g. Ito et al. 1999, 2006; Rutqvist et al. 2000; Raaen et al. 2006; Bunger et al. 2010; Gao et al. 2020). The main sources of uncertainty are the determination of the apparent reopening pressure, the residual aperture of the hydraulic fracture, the hydraulic fracture length compared to the near-borehole stress redistribution, and the estimation of the pore pressure. The difference between apparent and true reopening pressure, in particular, leads to an overestimation of $S_\mathrm {Hmax}$ (Ito et al. 1999, 2006; Gao et al. 2020). The main reason that the measured reopening pressure is higher than the true reopening pressure lies in the too high hydraulic compliance of the test equipments (Ito et al. 1999, 2006). Our wireline test system has a relatively low system compliance compared to conventional hydraulic fracturing systems with drillrods, but we were not able to quantify its effect on the reopening pressure determination. Consequently, our $S_\mathrm {Hmax}$ estimates should be understood as a possible range and as an upper estimate, since Eq. (3) likely overestimates $S_\mathrm {Hmax}$.

To quantify the $S_\mathrm {Hmax}$ uncertainty, uncertainties of the individual input variables in Eq. (3) were combined. The uncertainty of $S_\mathrm {hmin}$ is given by its standard deviation. For $P_\mathrm {r}$, we estimated the maximum error to be 1 MPa because of the graphical uncertainty in fitting the tangent line. We assigned a similar maximum error of 0.5 MPa for $P_\mathrm {p}$ to intervals where we conducted an overnight shut-in and an increased error of 1 MPa to all other intervals.

The $S_\mathrm {Hmax}$ magnitude of all four boreholes lies between 20.4 and 27.9 MPa (mean: $25.4 \pm 2.3$ MPa), which is close to the estimated magnitude of $S_\mathrm {v}$ ($\approx$ 26.5 MPa). The estimated uncertainty lies in the order of 2 to 4 MPa, which is considerably larger than the uncertainty of $S_\mathrm {hmin}$, and stems from the inclusion of many parameters and their individual uncertainties in Eq. (3).

When comparing the four boreholes, it is evident that SB1.1 and SB2.1 have lower average horizontal stress magnitudes than SB3.1 and SB4.1. In total, 12 out of the 19 mini-frac intervals yield $S_\mathrm {hmin}$ magnitudes between 14.5 and 16.5 MPa. The seven intervals with lower $S_\mathrm {hmin}$ magnitudes are all located in SB1.1 and SB2.1. The higher stress magnitudes in SB3.1 and SB4.1 could be due to the larger distance from the tunnel entrance associated with a higher overburden. The changing overburden and relatively shallow depth can cause local topographic effects on the stress field, which have to be investigated further (e.g. Liu and Zoback 1992; Chang and Jo 2015).

In addition to this inter-borehole variability, intra-borehole variations are evident. The minimum and maximum horizontal stress magnitudes are fairly consistent over depth in boreholes SB3.1 and SB4.1 (Fig. 15c, d), whereas SB1.1 and SB2.1 show larger variations (Fig. 15a, b). Between adjacent intervals, which are usually 4 m apart, in SB1.1 and SB2.1, $S_\mathrm {3}$ increases or declines by up to 3 MPa. No clear trend with increasing depth can be observed. A reason for the heterogeneity of the stress magnitude on the scale of a few tens of meters could be the presence of natural fractures (Fig. 15). The high stiffness contrast between the fractures and the stiff granitic rock mass might enhance stress variations (Chang and Jo 2015). Similarly, changes in the rock elastic properties can lead to stress concentrations in stiffer borehole sections. Another reason could be a combination of the stress perturbation, cooling and drainage around the tunnel. Numerical simulations in comparable settings showed that these effects can reach several tens of meters into the rock mass (Fu et al. 2018).

As previously mentioned, the pore pressure ranges from 2.0 to 5.6 MPa, which is significantly below hydrostatic values, which would be about 10 MPa at the depth of the BedrettoLab. Excavation of the tunnel and consequent drainage toward the unsealed tunnel have perturbed and lowered the pore pressure in its vicinity, as manifested by increasing pore pressure values with increasing distance from the tunnel. This is confirmed by pore pressure measurements in an about 100 m long borehole drilled from the Bedretto-Furka intersection at the northwestern end of the Bedretto Tunnel, which show pore pressures of 4.1 and 5 MPa at a distance of 30 and 51 m from the tunnel (Evans et al. 1999, personal communication).

To summarize, the mini-frac tests show that the stress regime in the vicinity of the BedrettoLab is transitional between normal and strike-slip faulting ($S_\mathrm {hmin}$ < $S_\mathrm {Hmax}$ $\le$ $S_\mathrm {v}$) with $S_\mathrm {hmin}/S_\mathrm {v} \approx$ 0.4–0.6 and $S_\mathrm {Hmax}/S_\mathrm {v} \approx$ 0.75–1.05. These results confirm the preliminary study by Ma et al. (2020).

## Concluding Remarks

We characterized the in situ stress state at the BedrettoLab using mini-frac tests in four short vertical boreholes, focusing on stress magnitudes. To get a reliable estimate of the least principal stress magnitude ($S_\mathrm {3}$) and a measure of its variability and uncertainty, we conducted an elaborated pressure transient analysis using five different methods. Four methods give consistent least principal stress estimates for the Bedretto granitic rock mass: the inflection point method, the bilinear pressure decay rate method, the fracture compliance method, and the jacking pressure from step-rate injection tests. The tangent method underestimates the stress magnitude and yields estimates that are below the frictional limit of the rock mass. The inflection point method gives a $S_\mathrm {3}$ estimate of $14.6 \pm 1.4$ MPa, which is very close to the mean jacking pressure of $14.8 \pm 1.5$ MPa. The bilinear decay rate shut-in pressure yields estimates that are on average 0.8 MPa lower than the inflection point estimates. The compliance method arrives at lower stress magnitudes and its application to pick the closure pressure is ambiguous because of variable pressure transient shapes, which are likely caused by the interaction of the hydraulic fractures with natural fractures. In some intervals, closure signals of natural fractures are absent during the first cycles but appear in the later ones, suggesting that the hydraulic fracture propagated and connected to natural fractures at some point.

The stress state, inferred from the mini-frac tests, in the vicinity of the BedrettoLab is transitional between normal and strike-slip faulting ($S_\mathrm {hmin}$ < $S_\mathrm {Hmax}$ $\le$ $S_\mathrm {v}$). The principal stress magnitudes are as follows: $S_\mathrm {hmin} = {11.2} - {16.4}\,$ MPa, $S_\mathrm {Hmax} = {20.4} - {27.9}\,$ MPa, $S_\mathrm {v} \approx {26.5}\,$ MPa, $P_\mathrm {p} = {2.0} - {5.6}\,$ MPa. The orientation of $S_\mathrm {Hmax}$ varies between $\hbox {N}\,{100}^{\circ }\hbox {E}$ and $\hbox {N}\,{120}^{\circ }\hbox {E}$. These findings agree well with the regional stress field and stress-induced failures (e.g., spalling and kinking) observed along sections of the Bedretto Tunnel. The local pore pressure values, obtained from extending shut-in times to up to 15 h, are below hydrostatic values. This indicates tunnel drainage effects that, together with cooling from the tunnel and its damage zone, can lead to complicated stress field changes around the Bedretto Tunnel.

The presented study focused on the estimation of the least principal stress magnitude from multiple short boreholes around the BedrettoLab, but several questions remain: What is the orientation of the hydraulic fractures farther from the borehole or after intersecting with natural fractures? What causes the inter- and intra-borehole stress variations? How does the stress state change at greater distance from the Bedretto Tunnel? Integrated stress analysis combining other independent stress indicators is warranted in our future work. This would complement our stress estimation based on mini-frac tests and could further constrain the stress uncertainty. Nonetheless, our results provided valuable ranges of the principal stress magnitudes that are necessary for generating numerical models and planning future experiment steps.

## Data Availability

The raw data and results are available at https://doi.org/10.3929/ethz-b-000463936 (Bröker et al. 2021).

## Appendix 1: The G-Function

The G-function was first introduced by Nolte (1979) and relates shut-in time to the duration of fluid injection:

4 $$\begin{aligned} G(\varDelta t_D,\alpha ) = \frac{4}{\pi }\left[ g(\varDelta t_D,\alpha )-g(0,\alpha )\right] \end{aligned}$$

with

5 $$\begin{aligned} g(\varDelta t_D,\alpha =0.5)= & {} (1+\varDelta t_D)\sin ^{-1}(1+\varDelta t_D)^{-1/2}+\varDelta t_D^{1/2} \end{aligned}$$

6 $$\begin{aligned} g(\varDelta t_D,\alpha =1)= & {} \frac{4}{3}\left[ (1+\varDelta t_D)^{1.5}-\varDelta t_D^{1.5}\right] \end{aligned}$$

where $\alpha$ is the power law exponent for fracture growth and $\varDelta t_D$ is the dimensionless pumping time, defined as $\varDelta t_D=\varDelta t/t_p$. Here $t_p$ is the duration of injection between the start of fluid injection and shut-in, and $\varDelta t$ is the time since shut-in.

The G-function builds upon several simplifying assumptions (Nolte 1979): a single planar fractures forms, the fluid pressure inside the fracture is constant during shut-in, fluid leak-off is describes by Carter’s leak-off model (Howard and Fast 1957), and the fracture growth scales with time according to a power law. Reasonable bounds for the power law exponent $\alpha$ are between 0.5 and 1.0, where $\alpha =1$ (Eq. 6) represents low leak-off or high fluid efficiency (fracture surface area after shut-in varies linearly with time), and $\alpha =0.5$ (Eq. 5) represents high leak-off or low fluid efficiency (fracture surface area varies with the square root of time after shut-in). The choice of $\alpha$ has no significant effect on the overall shape of the resulting pressure falloff curves, and we chose $\alpha$ = 1 as the granite is assumed to have very low permeability.

The G-function utilizes Carter’s leak-off model, which calculates the fluid leak-off by solving a one-dimensional pressure diffusion equation, assuming a constant fluid pressure inside the fracture and considering only flow perpendicular to the fracture walls. This model yields that fluid loss from the fracture is inversely proportional to the square root of time after shut-in (Howard and Fast 1957). It is taken into account that during fracture initiation and propagation the fluid volume lost through each newly created part of the fracture will tend to follow a square root of time evolution. The pressure decay of a fracture and borehole system with constant stiffness and Carter leak-off will be proportional to the square root of time (McClure et al. 2016; Detournay 2016), so it should give a straight line on a pressure versus square root of time plot.

Similarly, if all assumptions are valid, the cumulative fluid leak-off from the fracture after shut-in is linearly proportional to G time. Hence, the pressure versus G time plot should form a straight line before fracture closure, which means that the pressure derivative is constant (Eq. 7 after McClure et al. (2016)). The pressure derivative can be decomposed into fluid leak-off from the fracture and total system storage, described by the system storage coefficient $C_t$.

7 $$\begin{aligned} \frac{\hbox {d}P}{\hbox {d}G} = \frac{\hbox {d}V_{\mathrm{fr}}}{\hbox {d}G}\frac{1}{C_{\mathrm{t}}} = {\mathrm{const}}. \end{aligned}$$

The system storage consists of four parts: fluid compressibility in the borehole, fluid compressibility in the fracture, changes in borehole fluid volume, and changes in fracture fluid volume:

8 $$\begin{aligned} C_{\mathrm{t}} = c_{\mathrm{f}}V_{\mathrm{bh}} + c_{\mathrm{f}}V_{\mathrm{fr}} + \frac{\hbox {d}V_{\mathrm{bh}}}{\hbox {d}P} + \frac{\hbox {d}V_{\mathrm{fr}}}{\hbox {d}P} \end{aligned}$$

with $c_{\mathrm{f}}$ representing the water compressibility, $V_{\mathrm{fr}}$ the volume of fluid in the fracture, and $V_{\mathrm{bh}}$ the volume of fluid in the borehole. McClure et al. (2016) showed that the term $\mathrm {d}V_{\mathrm{fr}}/\mathrm {d}{P}$ can be expressed as $A/S_{\mathrm{fr}}$, fracture surface area divided by fracture stiffness, and that this term is the main factor causing the change in total system storage during fracture closure. The fracture stiffness is approximately constant as long as the fracture walls are not in contact and the fracture surface area is assumed to not change. Fracture closure leads to a stiffness increase, leading to an increase in the pressure derivative. The pressure derivative decreases again when the leak-off rate (first term on the in the middle of Eq. 7) reduces sufficiently enough to compensate for the stiffness increase, mostly because of the lowering fluid pressure inside the fracture. This pressure decline inside the fracture is a violation of the Carter leak-off assumptions, which makes all calculations using Eq. (7) an ideal theoretical case.

## Appendix 2: Semi-logarithmic Derivative

The first-order, semi-logarithmic time derivative, sometimes referred to as Bourdet derivative (Bourdet et al. 1989), is defined as:

9 $$\begin{aligned} \frac{\hbox {d}P}{\hbox {d}(\ln {G})} = G\frac{\hbox {d}P}{\hbox {d}G} \end{aligned}$$

The pressure data were resampled with a spline interpolation using a fixed number of time intervals regularly spaced in a logarithmic scale (Renard et al. 2009). This reduced noise in the pressure recordings, as it would otherwise have increased by calculating the derivative.

**Table 1:** Overview of the different methods used to estimate the $S_\mathrm {3}$ magnitude

Method	Plot used for estimation	Estimated pressure	Source
Inflection point	*P* vs. *t*	$P_\mathrm {si,inflection}$	Gronseth and Kry (1981) and Gronseth (1982)
Bilinear pressure decay rate	$\mathrm {d}P/\mathrm {d}t$ vs. *P*	$P_\mathrm {si,bilinear}$	Tunbridge (1989) and Lee and Haimson (1989)
Tangent	G $\cdot$ d*P*/dG vs. G time	$P_\mathrm {cl,tangent}$	Barree et al. (2007)
Fracture compliance	G $\cdot$ d*P*/dG vs. G time	$P_\mathrm {cl,compliance}$	McClure et al. (2014) and McClure et al. (2016)
Jacking pressure	*P* vs. *Q*	$P_\mathrm {jacking}$	Doe and Korbin (1987) and Rutqvist and Stephansson (1996)

**Table 2:** Determined pressure parameters in the four vertical stress measurement boreholes

Borehole	Depth (m)	$P_\mathrm {b}$ (MPa)	$P_\mathrm {r}$ (MPa)	$P_\mathrm {si,inflection}$ (MPa)	$P_\mathrm {si,bilinear}$ (MPa)	$P_\mathrm {cl,tangent}$ (MPa)	$P_\mathrm {cl,compliance}$ (MPa)	$P_\mathrm {jacking}$ (MPa)	$P_\mathrm {p}$ (MPa)	*T* (MPa)
SB1.1	14.5	24.3	15.8	$13.4 \pm 0.3$	$11.8 \pm 0.6$	–	$10.5 \pm 0.9$	14.6	–	8.5
	17.5	26.1	17.7	$16.1 \pm 0.6$	$14.4 \pm 0.8$	(7.6)	$12.3 \pm 0.2$	17.6	3.2	8.4
	19.9	20.1	12.8	$13.5 \pm 0.3$	$12.5 \pm 0.3$	–	–	13.2	–	7.3
	24.0	20.5	15.9$^{\mathrm{a}}$	$13.1 \pm 0.6$	$11.9 \pm 0.5$	$6.5 \pm 1.4$	$9.5 \pm 1.0$	14.5	3.9	8.1
SB2.1	12.5	25.5	19.2	$16.1 \pm 0.2$	$15.4 \pm 0.3$	$8.3 \pm 0.7$	$14.7 \pm 0.1$	16.6	2.0	6.3
	15.5	25.2	14.9	$13.5 \pm 0.4$	$12.3 \pm 0.4$	7.3	$10.8 \pm 0.3$	14.5	–	10.3
	17.5	24.6	14.8	$13.7 \pm 0.8$	$12.8 \pm 0.9$	$8.2 \pm 0.9$	$10.7 \pm 0.9$	–	2.7	9.8
	23.3	21.3	11.0	$11.2 \pm 0.3$	$10.4 \pm 0.3$	$7.0 \pm 0.2$	$9.3 \pm 1.4$	10.7	–	10.3
	25.2	20.7	17.9$^{\mathrm{b}}$	$13.9 \pm 1.5$	$13.6 \pm 1.4$	–	$13.3 \pm 2.4$	13.7	–	9.2
SB3.1	12.0	27.4	17.0	$16.4 \pm 0.4$	$15.8 \pm 0.7$	–	$15.6 \pm 0.8$	16.0	–	10.4
	16.0	27.0	16.4	$16.2 \pm 0.3$	$15.6 \pm 0.5$	–	$15.3 \pm 0.6$	15.6	–	10.6
	20.0	25.7	15.7	$16.3 \pm 0.4$	$15.7 \pm 0.6$	–	$15.5 \pm 0.7$	15.9	–	10.0
	24.0	19.1	13.7	$15.1 \pm 0.8$	$14.3 \pm 0.7$	–	11.5	15.2	–	5.4
	28.0	22.5	15.1	$14.7 \pm 0.3$	$13.8 \pm 0.5$	$10.7 \pm 0.4$	$12.9 \pm 0.3$	14.8	5.6	7.5
SB4.1	12.0	26.5	16.0	$15.1 \pm 0.5$	$14.4 \pm 0.9$	–	$13.9 \pm 1.4$	14.7	–	10.5
	16.0	26.3	16.0	$15.1 \pm 0.5$	$14.8 \pm 0.6$	–	$14.8 \pm 0.5$	14.6	–	10.3
	20.0	27.6	16.0	$15.4 \pm 0.4$	$14.9 \pm 0.5$	–	$14.7 \pm 0.8$	14.7	–	11.6
	24.0	25.5	14.4	$14.6 \pm 0.3$	$14.3 \pm 0.4$	$9.4 \pm 0.5$	$12.9 \pm 0.6$	(13.7)	2.6	11.1
	28.0	25.1	14.6	$14.5 \pm 0.3$	$14.0 \pm 0.2$	$10.1 \pm 0.5$	$13.5 \pm 0.2$	15.0	–	10.5

The mean and standard deviation are given if multiple cycles were evaluated. The values in brackets were determined with low confidence

$^{\mathrm{a}}$Likely overestimated, possible packer bypass

$^{\mathrm{b}}$Likely overestimated, fracture developed entirely below packer
